# Supplementary material for: Farnesol Boosts the Antifungal Effect of Fluconazole and Modulates Resistance in Candida auris through Regulation of the CDR1 and ERG11 Genes
Source: J Fungi (Basel). 2022 Jul 27;8(8):783. doi: 10.3390/jof8080783 (PMC9332773; doi:10.3390/jof8080783)
Supplement: Supplementary file 1 [file jof-08-00783-s001.zip › jof-1816135-supplementary.pdf]

Supplementary Material

Phenotype and genotype identification of *C. auris* isolates

The yeast strains were re-identified using different phenotypic and genotypic methods. For phenotype identification of strains, the Petri dish with CHROMagar Candida selective medium (Liofilchem, Italy) was inoculated by a volume of 20 μL of an overnight yeast culture. After 48 h cultivation at 37 °C, the morphology and staining of the colonies were considered. The result of cultivation is shown in Figure S1.

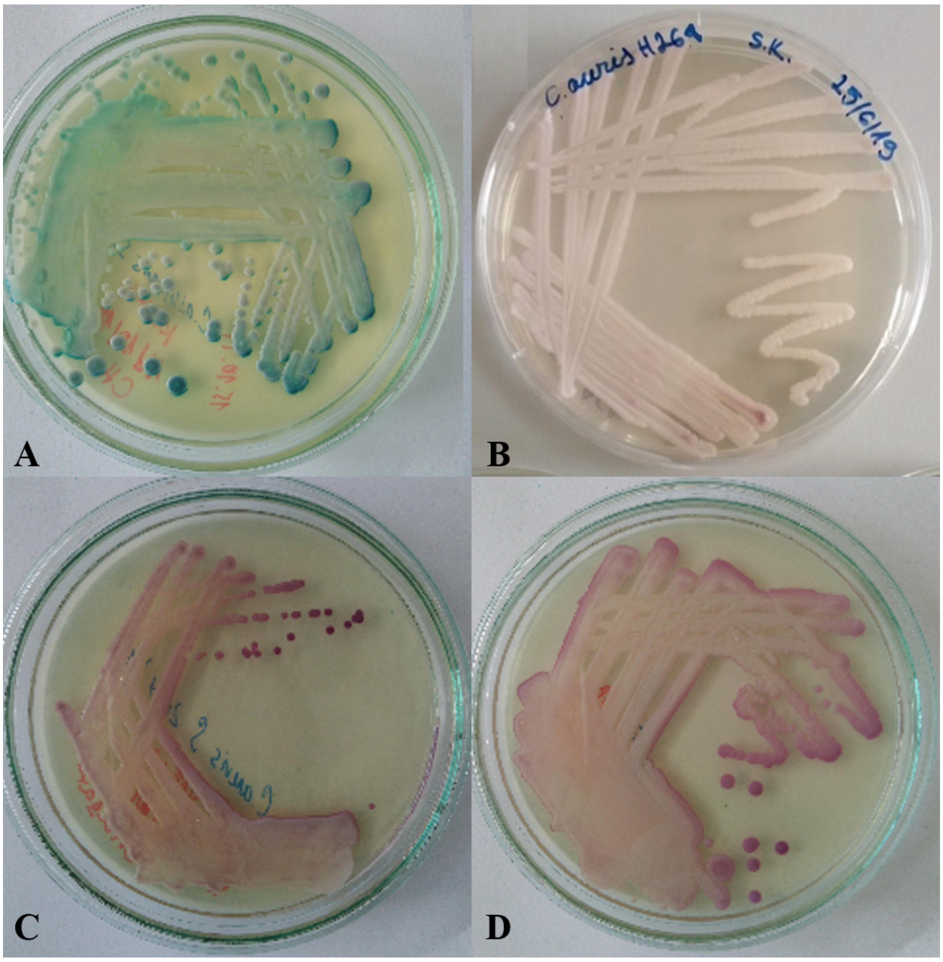

Figure S1. Phenotype identification of the isolates of Candida sp. A) *C. albicans* SC5314; B) *C. auris* H261; C) *C. auris* S; D) *C. auris* R

To accomplish genotype identification of strains, genomic DNA (gDNA) was isolated from yeasts by phenol extraction. One large loopful of microorganisms was transferred from the yeast peptone dextrose (YPD) agar (1% [w/v] yeast extract; 2% [w/v] peptone; 2% [w/v] dextrose; 2% [w/v] agar) into a 400 μL of TE solution (10 mM Tris-HCl, pH = 8.0; 1mM EDTA) in a microcentrifuge tube. An equal volume (400 μL) of the lower phase of phenol: chloroform: isoamyl alcohol (25:24:1) and approximately 200 μL of sterile glass beads (Sigma Aldrich, Germany) was added to the cell suspension. Subsequently, the suspension of cells was vortexed for approximately 5 min and then centrifuged (10 min, 4 °C, 14,000 RPM). After centrifugation, upper aqueous phase with dissolved nucleic acids (300 μL) was transferred using a micropipette to a 1.5 ml labeled Eppendorf tube. 30 μL of a 3 M NaOAc solution, pH = 5.2 and 900 μL of 96% ethanol were then added to the tube. After vortexing for 1 min, the tube was stored at -20 °C in the freezer for at least 20 min. Afterwards, the tube containing nucleic acids was centrifuged (10 min, 4 °C, 14000 RPM) and then the supernatant was discarded by using a micropipette. 500 μL of 70% ethanol was added to the obtained pellet and the sample in the tube was centrifuged again (5 min, 4 °C, 14000 RPM) in such a way as to maintain the same orientation of the tube in the centrifuge as in previous step. The supernatant was carefully removed and the tube with the pellet remained open in the sterile box for 15-20 min at room temperature. After drying the sample, 100 μL of Nuclease-free water was added to the pellet in an Eppendorf tube and the dissolved gDNA was gently mixed by vortexing. The concentration of isolated gDNA was measured using a NanoDrop Spectrophotometer, ND-1000 (NanoDrop Technologies, USA). The sample of gDNA was stored at -20 °C or used in Polymerase Chain Reaction (PCR). The properties and sequences of the oligonucleotide primers used in PCR technique are listed below (Table S1). Primers synthesized by Metabion AG, Germany, were diluted 1:10 in Nuclease-free water before use. Amplification of specific regions of DNA was performed in a 0.2 mL Eppendorf microtube with a total reaction volume of 20 μL. The samples were operated on ice. Reaction mixture (20 μL) consisted of 4 μL 5x FIREPol® Master Mix, 1 μL (0,01 – 10 ng/μL) gDNA, 0,5 μL Forward primer (1:10), 0,5 μl Reverse primer (1:10) and 14 μL Nuclease-free water. The PCR reaction was performed in an iCycler Thermal Cycler (BIORAD, USA). Nuclease-free water instead of gDNA was used as a negative control in each reaction. The PCR temperature cycling conditions were as follows: initial denaturation at 95 °C for 15 min; followed by 30 cycles of denaturation at 95 °C for 20 sec, *annealing at 54-66 °C for 30 s, and elongation at 72 °C for 1 min. The final cycle was followed by extension at 72 °C for 5 min.

*The temperature during the annealing phase was set 3-5 °C lower than the melting temperature (Tm) of the primers used in the reaction.

The gel electrophoresis in a 2% agarose gel was performed to visualize the fragments of DNA formed in the PCR reaction. The gel was prepared by boiling 1 g of agarose in 50 mL of 1x TBE buffer in a microwave oven until complete dissolution. An additional 5 μL of GoodView stain (Ecoli, Slovakia) was added to the agarose gel for nucleic acid visualization. Subsequently, the melted agarose was evenly poured into a gel casting tray containing a comb. After solidification, 10 μL of gDNA sample was pipetted into the gel wells, as well as BrightMax DNA Ladder (Canvax Biotech, Spain) and a negative control. Electrophoresis was performed in the presence of 1x TBE buffer at 100 V for 40-60 min (PowerPac™, Bio-Rad Laboratories Inc., USA). After separation, DNA fragments were visualized under ultraviolet light using an UV-Transilluminator MUV 21-312-220 (Major Science, USA) at a wavelength of 254 nm. The result of PCR is in electrophoretogram shown in Figure S2.

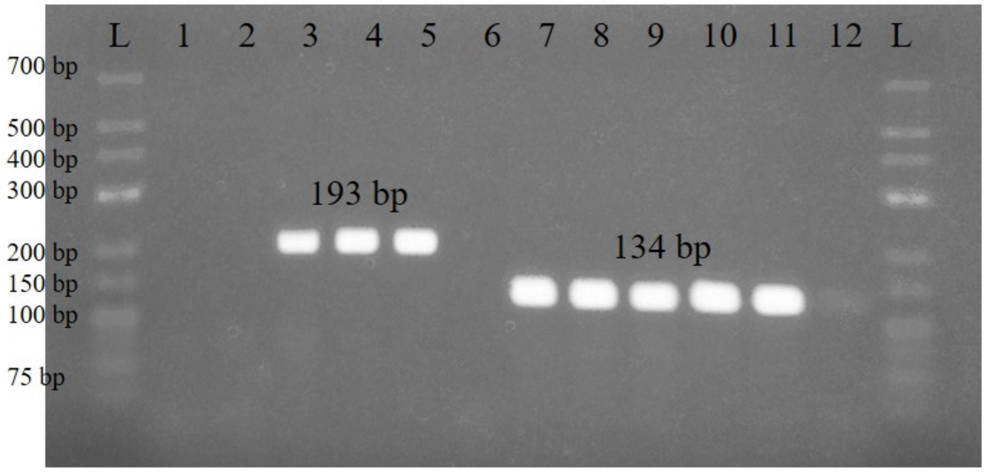

L – BrightMax DNA Ladder (25-700bp)
1. GQ_05701 C. albicans SC5314
2. GQ_05701 C. albicans CCY 29-3-164
3. GQ_05701 C. auris H261
4. GQ_05701 C. auris S
5. GQ_05701 C. auris R
6. GQ_05701 Negative control
7. 5.8S rDNA C. albicans SC5314
8. 5.8S rDNA C. albicans CCY 29-3-164
9. 5.8S rDNA C. auris H261
10. 5.8S rDNA C. auris S
11. 5.8S rDNA C. auris R
12. 5.8S rDNA Negative control

Figure S2. Electrophoretogram of PCR products for genotype identification of *C. auris* and *C. albicans*.

Table S1. List of oligonucleotide sequences used in this study

Gene	Forward primer	Reverse primer	Reference
RDN5.8S rDNA	GGATCTCTTGGTTCTCGC	CGCTCAAACAGGCATGC	Ruit-Gaitán et al. 2017
GQ_05701	GCAGCACTCGTGAGAGAACT	GGCTGGTTCTCCTGCTCATT	
ACT1	GAAGGAGATCACTGCTTTAGCC	GAGCCACCAATCCACACAG	Rybak et al. 2019
CDR1	GAAATCTTGCACTTCCAGCCC	CATCAAGCAAGTAGCCACCG	
CDR2	GTCAACGGTAGCTGTGTG	GTCCCTCCACCGAGTATGG	
MDR1	GAAGTATGATGGCGGGTG	CCCAAGAGAGACGAGCCC	
ERG11	GTGCCCATCGTCTACAACCT	TCTCTCTGCACAGCTCGAAA	Bhattacharya et al. 2019

References:

Rybak JM, Doorley LA, Nishimoto AT, Barker KS, Palmer GE, Rogers PD. Abrogation of Triazole Resistance upon Deletion of *CDR1* in a Clinical Isolate of *Candida auris*. Antimicrob Agents Chemother. 2019, 63(4):e00057-19. doi: 10.1128/AAC.00057-19

Bhattacharya S, Holowka T, Orner EP, Fries BC. Gene Duplication Associated with Increased Fluconazole Tolerance in Candida auris cells of Advanced Generational Age. Scientific Reports. 2019, 9(1)

Ruiz-Gaitán AC, Fernández-Pereira J, Valentin E, Tormo-Mas MA, Eraso E, Pemán J, de Groot PWJ. Molecular identification of Candida auris by PCR amplification of species-specific GPI protein-encoding genes. Int J Med Microbiol. 2018, 308(7), 812-818
